# Updates in Urologic Robot Assisted Surgery

**DOI:** 10.12688/f1000research.15480.1

**Published:** 2018-12-18

**Authors:** Anojan Navaratnam, Haidar Abdul-Muhsin, Mitchell Humphreys

**Affiliations:** 1 Department of Urology, Mayo Clinic in Arizona, Phoenix, AZ, 85054, USA

**Keywords:** Robotic surgical procedures, Ureteroscopy, Laparoscopy, Prostate ablation, Near-infrared imaging

## Abstract

Modern robotics is an advanced minimally invasive technology with the advantages of wristed capability, three-dimensional optics, and tremor filtration compared with conventional laparoscopy. Urologists have been early adopters of robotic surgical technology: robotics have been used in urologic oncology for more than 20 years and there has been an increasing trend for utilization in benign urologic pathology in the last couple of years. The continuing development and interest in robotics are aimed at surgical efficiency as well as patient outcomes. However, despite its advantages, improvements in haptics, system size, and cost are still desired. This article explores the current use of robotics in urology as well as future improvements on the horizon.

## Introduction

Since the initial introduction and subsequent adoption of the da Vinci™ robotic system (Intuitive Surgical Inc., Sunnyvale, CA, USA) in the late 1990s, there have been increasing improvements in magnification, three-dimensional viewing, and maneuverability that enable surgeons to complete certain surgical operations that once were thought to be relegated to open surgeons ^1, 2^. Urologists have a proven record of early adoption of new technologies (such as lasers and flexible endoscopy) that enable interventions for patients in a less-invasive manner.

Urologists championed robotics initially for radical prostatectomy starting in 2001 ^3^. Despite randomized trials suggesting minimal improvements in functional outcomes with the introduction of robot-assisted radical prostatectomy (RARP), 90% of radical prostatectomies in the US continue to be done robotically ^1, 4^. Moreover, there is an increasing trend in the use of robotics in the surgical treatment of other indications such as reconstructive urology and the management of urolithiasis and benign prostatic hyperplasia (BPH). As such, there is a continued demand for further research and development to improve robotic technology to a degree where it is superior to traditional surgical approaches despite the global resource and economic challenges.

Current barriers to overcome are haptic feedback, size and footprint of the robotic system, and inability to quickly switch between instruments during a procedure (see Table 1) ^5^. The purpose of this report is to explore the latest robotic technology available and what is on the horizon for urologic surgery.

**Table 1.** Summary of key urological robotic technology.

Device name	Console	Telescope	Robotic arms	Clinical applications
da Vinci™ Xi (Intuitive Surgical Inc., Sunnyvale, CA, USA)	3D binocular viewing console, Endowrist™ finger controls, foot pedals for clutching and application of energy	3DHD 8 mm camera	Four robotic arms hanging from an overhead boom system allowing multiquadrant access	Pelvic surgery, retroperitoneal surgery, intracorporeal reconstruction
da Vinci™ (Intuitive Surgical Inc.) SP 1098	As above Additional foot pedal to allow movement of robotic arms in unison	3DHD 12 mm articulating camera	Boom-mounted single robotic arm with single 25 mm robotic port 6 mm articulating robotic instruments allowing triangulation	Single-port surgery Cadaveric studies for prostatectomy, cystectomy, and partial nephrectomy complete US Food and Drug Administration-approved
Revo-i™ (Meere Company, Yongin, Republic of Korea)	3D binocular viewing console, wristed instrument control with hand clutch Foot pedal camera clutch	3DHD 10 mm camera	Four robotic arms mounted to boom Instruments are 7.4 mm in diameter	Only clinical study has been with Retzius-sparing radical prostatectomy
Roboflex Avicenna™ (Elmed Medical Systems, Ankara, Turkey)	Adjustable seat with armrests for surgeon Joystick controls Touchscreen to modify speed of deflection, adaption to mode of ureterorenoscope, advancement and retraction of laser fiber, adjustment of irrigation flow rate Two foot pedals control laser and fluoroscopy	Off-the-shelf existing digital ureteroscope	Robotic manipulator which holds ureteroscope Endoscope stabilized by two holders	Flexible ureteroscopy
Auris robotic endoscopy system (ARES™) (Auris Surgical Robotics, Redwood City, CA, USA)	Video game-like hand controller with electromagnetic-generated real-time navigation	Remote driving fully incorporated flexible digital endoscope	Three robotic arms including camera with multiple channels allowing the passage and control of laser fiber and irrigation	Flexible ureteroscopy
AquaBeam System™ (Procept BioRobotics, Redwood Shores, CA, USA)	Stand-alone with keyboard and touchscreen An input for transrectal ultrasound which maps and plans subsequent prostate resection	22F Rigid Hopkins Cystoscope	Articulating robotic arm that delivers high-pressure saline in longitudinal and rotational movements	Benign prostate hyperplasia

3D, three-dimensional; 3DHD, three-dimensional high-definition.

## Robotic laparoendoscopic single-site surgery

In 2008, the first robotic laparoendoscopic single-site surgery (RLESS) series in the urological literature was published with four patients undergoing radical prostatectomy ^6^. Since then, a number of studies have investigated the application of this technique to a number of other urological procedures—including simple prostatectomy, partial nephrectomy, and pyeloplasty—with varying degree of success ^7, 8^. However, widespread adoption of RLESS has been limited by issues such as instrument clashing and limited ergonomics for the bedside assistant.

**Figure 1.**
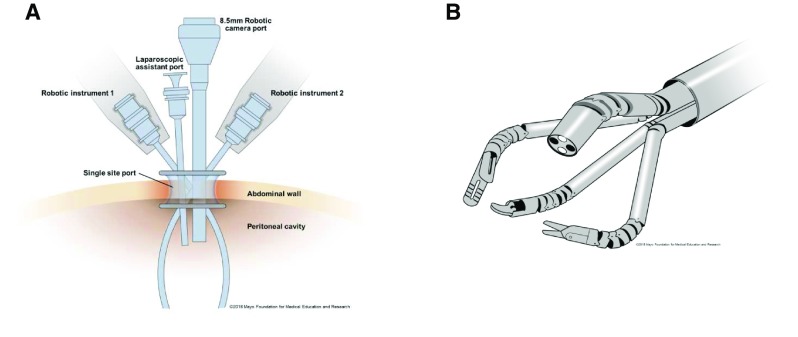
Demonstration of single site versus single port. ( **A**) Single-site configuration with separate robotic and assistant port entering through a common multichannel port. This is the “chop-stick” configuration of instruments and robotic arms. ( **B**) The single-port schematic with robotic instruments introduced through a single-channel robotic port. Instruments are articulated. *Used with permission of Mayo Foundation for Medical Education and Research. All rights reserved.*

Single-port (SP) surgery should be differentiated from single-site (SS) surgery. Both use a single skin incision; however, SP involves only a small incision through the fascia through which a single-channel port is introduced that conveys all of the robotic working instruments, whereas SS requires a larger skin incision that accommodates the introduction of multiple instruments through a multichannel working port ^2^ ( Figure 1).

The current US Food and Drug Administration (FDA)-approved da Vinci™ platform is an SS system that uses the Si™ system. A multichannel port is used through which different robotic ports can be placed. Various ports have been used, including the GelPort™ (Applied Medical, Rancho Santa Margarita, CA, USA), TriPort™ and R-Port (Advanced Surgical Concepts, Bray, Ireland), and SILS™ port (Covidien, Minneapolis, MN, USA). Most studies to date have reported issues with loss of insufflation during robotic cases as well as clashing of instruments and difficulty integrating with the bedside assistant during the case ^2, 8, 9^. Through the access port, an 8.5 mm robotic camera, two curved semi-rigid robotic instruments, and a rigid laparoscopic assistant port are inserted. The robotic instruments are semi-rigid to allow passage of the instruments through the curved cannulas ^10^. These instruments do not have the seven degrees of freedom that is standard in most da Vinci™ robotic instruments.

The “chop-stick” technique was developed to minimize external clashing of robotic arms ( Figure 1A). This involves increasing the external distance of robotic instruments and having them cross to the contralateral side of the body after bypassing the fascia ^2, 10^. Same-sided hand-eye control is maintained through assignment software that enables the surgeon’s ipsilateral hand to control the ipsilateral instrument on the screen. The main limitation of this technique is intra-abdominal clashing at the level of the fascia. Additionally, movement of the instruments is an “all or none” phenomenon with the inability to move one instrument without moving the others. Although needle drivers on this system have articulated wrists, the remaining instruments are not articulated with the seven degrees of freedom associated with the standard robotic platform. Hence, the instruments act more like laparoscopic instruments with movements in one plane.

SS surgery using the above configuration has been implemented in a small series for a wide array of urological procedures, including radical prostatectomy, simple prostatectomy, and partial nephrectomy ^6– 8^. There have been no randomized studies in the urologic literature to date, but in their study for RLESS cholecystectomy, Pietrabissa *et al*. demonstrated no difference in pain compared with standard four-port laparoscopic cholecystectomy but improved cosmetic satisfaction ^11^. For partial nephrectomy, it has been demonstrated that RLESS was less likely to achieve the trifecta of warm ischemia time of less than 20 minutes, negative surgical margins, and no intraoperative complications when compared with standard robot-assisted partial nephrectomy (RAPN) ^2^. Radical prostatectomy has, via RLESS, been demonstrated in the hands of experienced robotic surgeons with comparable outcomes and without serious complications ^6, 12^.

The main limitation of the SS robotic approach is that the original da Vinci™ platforms were not engineered specifically for SS surgery. This has led to the development of an SP system. The latest SP system is the da Vinci SP™ (Intuitive Surgical Inc.) and was released after FDA approval in May 2018. This was based on the third-generation prototype, of which preclinical studies had been conducted, called the SP 1098. A single 25 mm port accommodates a 12 mm articulating camera, three curved semi-rigid robotic instrument ports, and an 8 mm straight assistant port ^13^ ( Figure 1b). An improvement with the SP system is improved instrument clutching. Unlike in previous systems, the surgeon is able to clutch and pivot the instrument arm about its remote center without moving each additional instrument ^13^. An instrument is able to remain stationary in one location while the remaining instruments are reoriented to work in a separate working space ^13^. This improves the available workspace in which to operate. Additionally, all working instruments can be articulated to align this system with the standard da Vinci™ platform.

The application has been limited mainly to preclinical cadaveric studies for retroperitoneal partial nephrectomy and perineal radical cystectomy and extended pelvic lymphadenectomy ^13, 14^. These studies demonstrated improved cosmesis from a single incision, and the authors commented on the improved articulation and triangulation that overcame obstacles seen with older SS platforms ^10^. Both of these studies demonstrated the feasibility to complete both procedures with improved ergonomics compared with the previous SP platform; however, there were some difficulties experienced with the intracorporeal urinary diversion in the cystectomy study.

A similar console-based robotic SP platform is the Single Port Orifice Robotic Technology (SPORT™) surgical system (Titan Medical Inc., Toronto, ON, Canada). A single-arm bedside cart is controlled by a surgeon at a remote console with hand controllers and foot pedals. Rather than using a binocular viewing console, the surgeon views the surgery on a three-dimensional high-definition (3DHD) flat screen. It is an SP device that is deployed through a 25 mm robotic port. The instruments are “multi-articulated”, but it is unclear what degrees of motion they allow ^15^. Titan Medical had previously developed the Amadeus RSS™ but stopped development in 2013 ^3^. This platform is still in the preclinical stages of testing and has yet to be FDA approved.

Creating a specialty-purposed engineered SP robotic system appears to have improved the common hurdles previously experienced by surgeons using off-the-shelf adapted robotic platforms. Although SP and SS platforms may improve patient cosmesis and satisfaction outcomes ^11^, further clinical studies are required to assess whether such techniques will impact pathological outcomes with decreased morbidities.

## Image-guided robotic urologic surgery

### Near-infrared fluorescence imaging

Image-guided surgery is very well established in urology and its recent application to robotic surgery aims to augment or enhance surgery and outcomes for patients. The most common application is the use of near-infrared fluorescence (NIRF) imaging during robotic surgery which assists surgeons by identifying vascular anatomy with better accuracy than the naked eye. The da Vinci Si™ and Xi™ (Intuitive Surgical Inc.) platforms are both equipped with an NIRF technology (Firefly™, Intuitive Surgical Inc.) to allow toggling between normal and enhanced images during surgery. Indocyanine green (ICG) dye is an FDA-approved water-soluble dye that is used as the marker that the NIRF camera detects. It is favorable, as it (1) is confined to the vascular compartments after intravenous administration, (2) has a short plasma life (3–5 minutes), and (3) has a low tissue toxicity of 0.34% ^16, 17^. The robotic camera uses an 805 nm wavelength laser toward the target anatomy. This provokes detectable photon emission at 830 nm which is detected by the NIRF component of the robotic camera ^16^. Patented software on the da Vinci™ superimposes a green image indicating the presence of ICG in blood vessels. The cost of NIRF appears to be minimal on a per-case basis given that the current robotic platforms have this technology built in. It has been estimated that NIRF adds $80 to $100 a case; however, these figures are based on small numbers and would vary between institutions ^17^.

**Figure 2.**
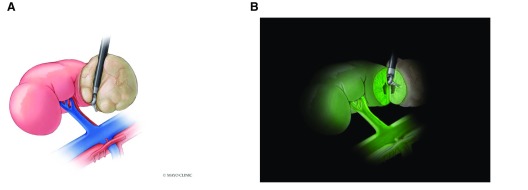
Diagram demonstrating concept of using indocyanine green (ICG) and near-infrared imaging. ( **A**) Right renal pedicle with artery and vein with white light and upper pole tumor. ( **B**) Fluorescence (green) after injection with ICG with near-infrared imaging. Green surgical margin compared gray region of tumor lacking ICG uptake. *Used with permission of Mayo Foundation for Medical Education and Research. All rights reserved.*

There have been numerous applications of NIRF across all forms of robotic surgery. RAPN for renal cell carcinoma has been the most widely investigated procedure. In healthy renal parenchyma, ICG binds to an enzyme transporter, bitranslocase, and appears isofluorescent when perfused with ICG-laden blood ^16^. Tumors are deficient in this transporter and appear hypofluorscent ^16, 17^ ( Figure 2). This has the potential benefit of improved outcomes regarding nephron sparing of normal tissue and improved oncological margins. Most studies to date have primarily investigated the ability to perform selective arterial clamping and impact on post-operative renal function. A multi-institutional study of 34 patients using selective arterial clamping compared with main renal artery clamping with the use of NIRF demonstrated that almost 80% were able to undergo selective arterial clamp RAPN with no difference in comparable blood loss or margin status. Improved short-term improvements in estimated glomerular filtration rate (eGFR) were noted at 10 to 30 days ^18^. Similar results have been demonstrated in other studies; however, the impact on long-term changes in eGFR based on super selective versus main arterial clamping appears to be minimal ^16^.

The use of ICG in RARP has also been investigated to aid in neurovascular bundle identification and lymph node dissection. Bates and Patel demonstrated that 30% of prostatic neurovascular dissections may not correctly identify the “landmark” artery that requires preservation ^16^. The correlation to improvement in erectile function and continence has not yet been investigated.

Lymph node staging of prostate cancer has always been limited by the lack of reliable preoperative imaging as well as the ability to address all possible landing zones for prostate cancer metastases intraoperatively. Recent utilization of positron emission tomography (PET) scans using various radiolabeled markers such as prostate-specific membrane antigen (PSMA) has aimed to address this gap in staging imaging. However, marrying this with intraoperative visualization of lymph nodes and decision making is the next step in the evolution and integration of these modalities. Initial studies investigated the use of ICG radiolabeled with the nanoparticle 99mTc-NanoColl along with the use of a laparoscopic gamma probe and NIRF camera to identify sentinel nodes ^17^. More recently, pilot studies have investigated the use of ICG to perform sentinel lymph node biopsies during RARP with the use of Firefly™, yielding a sensitivity and specificity of 100% and 75.4%, respectively ^19^. ICG bound to PSMA appears to be the next possible marker to assist with intraoperative lymph node dissection; however, this is still in the preclinical stages of investigation ^20^. Given increased use of PSMA PET imaging for the staging of prostate cancer ^21^, such developments would allow surgeons to correlate preoperative and intraoperative findings and give a better sense of the accuracy of these enhanced imaging modalities.

NIRF can also be used in reconstructive surgery by demonstrating perfusion of the urothelium. Although the exact mechanism of this is not clear, injection of ICG has allowed the identification of ischemic segments of the ureter in pyeloplasty, ureteral reimplantation, and ureteroureterostomy. In this setting, the poorly perfused ischemic segment appears dark compared with normal urothelium, aiding the identification of healthy tissue for reconstruction ^22^. NIRF has also been used in cystectomy to identify the mesenteric vessels and to confirm perfusion of distal ureteral stumps during intracorporeal diversion. Manny and Hemal were able to identify mesenteric arcades in eight out of eight patients undergoing intracorporeal diversion with no ischemic complications on short-term follow-up ^23^. Further studies are required to confirm these findings, but, given the low toxicity and short onset of action, NIRF appears to be a useful tool during reconstructive urological surgery.

### Augmented reality

Surgeons have traditionally relied on their evaluation of preoperative high-resolution cross-sectional imaging, knowledge of relevant anatomy, and their previous experience when embarking on complex surgical procedures ^24^. Augmented reality (AR) involves superimposing models specific to the patient onto the surgical field to assist with intraoperative decision making ^24, 25^. The two stages by which AR provides potential advantages are by facilitating the accurate anatomic relationships of the target organ and by facilitating correct tumor resection, ensuring negative surgical margins ^25^.

Early experience centered on laparoscopic prostatectomy involved the use of simultaneous transrectal ultrasound with navigational fiducial markers to overlay images of the prostate and neurovascular bundles ^26^. This evolved to utilization of magnetic resonance and computed tomography (CT) images by software platforms to create maps which can be superimposed on real-time intraoperative images. Porpiglia *et al*. recently demonstrated the accuracy of the superimposed AR image with the endoscopic view during RARP ^27^. Their primary endpoint demonstrated an accuracy of 100% for identification of the index lesion in the prostate ^27^. AR was able to identify extracapsular extension at the neurovascular bundles in 73.3% of cases which were positive for cancer. With regard to accuracy of image registration, they compared this with both the wet specimen and cross-sectional images of the specimen and demonstrated a surface area mismatch ranging from 1 to 5 mm with a volumetric mismatch ranging from 20% to 30% ^27^.

Most studies investigating the use of AR in RAPN have been conducted in the *ex vivo* setting. These studies demonstrated feasibility and safety of the technology in partial nephrectomy ^25^. There were conflicting results on the accuracy of AR in partial nephrectomy. Herrell *et al*. reported improved nephron sparing with reduced normal parenchyma-to-tumor ratios in *ex vivo* tumors resected with AR assistance ^28^. However, other studies have demonstrated target registration errors ranging from 1 to 5 mm ^25^. These differences are pertinent to partial nephrectomy when margin status may be critical depending on the tumor type as more aggressive partial nephrectomies are carried out. Herein lies the main limitation of AR: the camera’s ability to track organs accurately and quickly enough after image registration. Owing to surgical dissection, organ manipulation, and cardiorespiratory movement, the superimposed AR images do not make dynamic changes or tissue deformation ^25^.

One method by which the above limitations may be curbed is navigational software for organ tracking during surgery. Electromagnetic-guided collimators have been used by radiation oncologists to track the natural movements of the prostate during radiation treatment ^29^. There has been some application in experimental models of this technique with AR; however, it requires implantation of magnetic trackers into the target tissue which may compromise safe oncologic margin control. There are also questions of how these markers would interact with robotic instruments or other ferromagnetic objects in the operating room.

Image-guided surgery has the potential to overcome the lack of haptic feedback by providing additional visual cues to assist surgeons. Simple techniques such as the utilization of NIRF do not appear costly and can improve intraoperative decision making, translating to better oncological and functional outcomes in urological robotic surgery. AR provides the next step to image assistance during surgery but still requires refinement of the technology to make it more precise and dynamic intraoperatively.

## Robot-assisted ureteroscopy

The use of flexible ureteroscopy (FURS) has increased exponentially in recent times because of improvements in equipment miniaturization, deflection, and digital visualization. It is becoming the most commonly used treatment for patients with urinary stones with increased application in large complex renal calculi where percutaneous nephrolithotomy and extracorporeal shockwave lithotripsy may have previously been deployed ^30, 31^. However, the application of FURS in this setting can be limited by suboptimal vision, maneuverability of the ureteroscopy, fragility of the instrumentation, and poor surgeon ergonomics. In fact, one-third of endourologists have reported hand–wrist problems as a result of FURS ^32^.

The initial application of robotics in ureteroscopy was investigated in 2011 using the Sensei X™ robotic catheter system (Hansen Medical Inc., Mountain View, CA, USA) ^33^. This was originally designed for cardiac catheterization. The system is controlled by an electromagnetic slave guided by a computerized master ^5^. The surgeon controls the end effector with an external console and a joystick ^5, 33^. The flexible ureteroscope is placed in the flexible robotic catheter system which is capable of 275° of deflection ^5^. In their study, Desai *et al*. demonstrated completion of the procedure in 18 patients with a mean stone size of 11.8 mm and a mean total operative time of 91 minutes ^33^. There were no intraoperative complications while 89% of patients had complete stone clearance at 3-month follow-up imaging. The system rated highly regarding visual analogue scoring for navigation stability and ability to fragment stones ^33^.

This study was followed up in a multi-institutional European study with a robot specifically designed for ureteroscopy, the Roboflex Avicenna™ (Elmed Medical Systems, Ankara, Turkey). The design of this robotic system was similar as above with placement of a ureteroscope into the robotic deflector controlled at a surgeon console with a joystick. This system had additional benefits of control interfacing with fluoroscopy and the ability to advance laser fibers and control the irrigation flow rate. A total of 74 patients were treated with a mean stone size of 13 mm, operative time of 74 minutes, and stone clearance at 3 months of 80% ^32^. The authors suggested that their system was more intuitive compared with the Hansen Medical system because of their faster stone location time. The seven surgeons included in the study noted improved ergonomics with the robotic platform compared with classic FURS.

Advantages of robot-assisted ureteroscopy include improved efficiency of dusting or fragmentation with greater ability to control the scale of deflection during the procedure depending on stone size ^33^. Much like other robotic technologies, it hopes to become an enabling technology making complex procedures available to the masses. Features such as display of the degree of endoscope deflection and fine control of this may translate to less deterioration of flexible ureteroscopes which often have a limited lifespan of 20 to 50 uses ^32^. Additionally, there is potential for improved efficiency owing to the reduced need for assistants and time for changing of instruments such as baskets and laser fibers. There is the added advantage of less radiation exposure to the surgeon given the potential for the master unit to be located well away from the radiation field during fluoroscopy.

The above two systems require placement of a standard flexible ureteroscope into their robotic platform and are not an “all-in-one” system with the ureteroscope incorporated into the system. The Auris robotic endoscopy system (ARES™) (Auris Surgical Robotics, Redwood City, CA, USA) is a teleoperated endolumenal system that has been FDA-approved since 2016 for bronchoscopy ^5^. The system is made of a bedside cart with three robotic arms which include a camera that has multiple channels allowing the passage of instruments and irrigation fluid. It relies on electromagnetic generated navigation to control the inbuilt ureteroscope. Preclinical trials were recently presented at the American Urological Association in Boston in 2017. If performance is similar, the main obstacle to implementation may be cost, particularly since the other two systems are able to use equipment already available in the majority of institutions.

## Robot-assisted transurethral prostate treatment

Aquablation is a novel minimally invasive water ablative therapy for the resection of prostate tissue for the treatment of lower urinary tract symptoms as a result of BPH. Aquablation combines real-time image guidance via transrectal ultrasound with an automated robotic arm that uses a high-pressure saline jet to ablate prostate tissue ^34^. The AquaBeam™ (Procept BioRobotics, Redwood Shores, CA, USA) was approved in December 2017.

The system consists of three main components: (1) the console, (2) the robotic hand-piece, and (3) the single-use resection probe ^34^. After manual inspection of the bladder with a usual 22F rigid cystoscope, the waterjet firing probe is introduced to the bladder neck. The articulating arm is then anchored followed by insertion of the transrectal ultrasound to map the prostate and plan the subsequent resection ^34^. There is then a period of image capture using the ultrasound to map to the prostate and plan the subsequent resection. The ablation then begins with high-pressure, non-heated normal saline delivered with rotational and longitudinal movements to accurately resect the prostate ^34^. Hemostasis is achieved with focal electrocautery or low-pressure inflation of a foley balloon catheter in the prostatic fossa at the end of the procedure and continuous bladder irrigation ^35^.

The recent WATER study was a multi-institutional randomized controlled trial comparing Aquablation™ (Procept BioRobotics) with transurethral resection of the prostate (TURP), and the primary endpoints were improvement in the International Prostate Symptom Score (IPSS) at 6 months after procedure and safety of the operation. A total of 177 patients were enrolled in the study. There was no difference in IPSS, Qmax, quality-of-life score, or post-void residual at 6 months. Although mean operative times were similar between the two groups, resection time was significantly lower in the Aquablation™ group compared with TURP (4 and 27 minutes, respectively) ^35^. The safety profile of the former was also better compared with TURP. The primary safety endpoint measured included Clavien–Dindo grade 2 to 5 events and any grade 1 events that resulted in permanent disability, including incontinence and erectile and ejaculatory dysfunction. Adverse events were significantly lower in the Aquablation arm compared with TURP (25.9% versus 41.5%, *p* <0.02) ^35^. Only one patient in the intervention arm required a blood transfusion compared with none in the TURP arm. In particular, there was improved efficacy in larger glands (50–80 mL) which can be technically challenging for urologists because of increased vessel density and multinodularity of the glands. A recent follow-up study also confirmed the feasibility of this technology for large prostate glands (80–150 mL) with resection times of only 8 minutes; however, blood transfusion was required perioperatively in 5.9% of cases ^36^.

This technology highlights the potential for a robotic platform to improve the efficiency of surgery for BPH. The promising results for large glands regarding resection and safety may help reduce the morbidity often associated with operating on these complex patients. Further studies are required to investigate the durability of these initial results as well as cost analysis. However, with significantly lower resection times, Aquablation™ could provide an efficient means of addressing a common pathology in urology.

## Other robot technology on the horizon

### Competition to current robotic surgical systems

The Revo-i™ (Meere Company Inc., Yongin, Republic of Korea) is a robotic surgical system that has been in development in South Korea since 2006. In 2017, it received Korean Food and Drug Administration approval for clinical use ^37^. It is similar to the da Vinci™ S and Si platforms with an open console using a 3DHD telescope and a separate four-arm mounted cart ^3^.

Chang *et al*. recently reported on the initial results of the use of this robot for Retzius-sparing RARP in 17 patients ^37^. The authors were able to complete all surgeries successfully without the need to convert to use of the standard da Vinci™ or open. Their operative times and estimated blood loss were comparable to those of previously reported randomized trials studying RARP; however, they did report a blood transfusion rate of 17.6% ^4, 37^. There was an overall positive surgical margin (PSM) rate of 23.5% (pT2 PSM 18.8% and pT3 100%), which is comparable to previously reported rates for Retzius-sparing RARP. At 3 months, the continence rate (zero pads) was 70.6%, which again was similar to previously reported results from this technique.

This system has demonstrated early safety and feasibility in the clinical application of Retzius-sparing radical prostatectomy. The true test will be seen if these results hold true with a larger cohort of patients and in other operations. This type of system, comparable to da Vinci™, will hopefully lead to a marketplace adjustment and a global decrease in the expense of robotic systems.

### Improving haptics

Haptic feedback involves the surgeon’s having the ability to sense the degree of force they are applying to tissue. The current da Vinci™ system relies on visual cues as a surrogate for “pseudo-haptics” to assess the degree of tension on tissues. The Senhance™ (TransEnterix, Morrisville, NC, USA) robotic platform is a console formerly known as Telelap ALF-X, which recently obtained FDA approval for use in minimally invasive surgery. The design incorporates a remote 3DHD display coupled with an infrared eye-tracking camera control which centers the image at the point of focus for the surgeon ^5^. Haptic feedback is scaled at 1:1 through the laparoscopic handles at the console ^5^. The technology is patented; however, the tactile force feedback translates sensation from an instrument’s distal end to the surgeon’s hand. Use in gynecological surgery has demonstrated comparability to standard laparoscopy. Surgeons were able to safely complete hysterectomy and bilateral salpingo-oopherectomy in 10 obese patients and did not need to convert to open or standard laparoscopy ^38^. Another study, in 45 patients undergoing colonic resection for benign disease, demonstrated that the system was feasible and safe ^39^. One significant advantage of the system was the lack of instrument clashing, and this was owing to completely independent robotic arms. The instruments are not wristed and are limited in their movement to 90° articulation and 360° rotation ^39^.

The Versius Robotic System (Cambridge Medical Robotics Ltd., Cambridge, UK) is a lightweight robotic platform where five separate robotic arms are controlled by an operator console with a joystick. The surgeon views the operation on a screen while wearing 3DHD glasses. The technology also provides force haptic feedback delivered to the surgeon controller. FDA approval is expected for 2019 with release in Europe in 2018. The CEO of the company was recently quoted as saying the company’s aim was to reduce whole lifetime cost in the hospital by up to 40% ^40^.

### Miniaturization of equipment

A common criticism of the current da Vinci™ system is the bulk associated with the console, bedside, and vision carts. This often requires larger operating rooms for deployment and storage. Downsizing of equipment would be a valuable modification in future robots. The Virtual Incision Corporation (Pleasanton, CA, USA), in collaboration with the University of Nebraska Medical Center, Omaha, has developed the Miniature In Vivo Robot (MIVR™, Virtual Incision Corporation and University of Nebraska Medical Centre, Omaha, NE, USA), which allows insertion and maneuverability completely inside the peritoneal cavity through a single incision. It is composed of two arms with a flexible robotic camera and can provide multiquadrant access to the abdomen ^5^. It has the key advantage of minimal extracorporeal equipment, resulting in less labor-intensive storage and maneuverability. This technology is still in the preclinical stage and is not FDA-approved.

### Autonomous robot systems

True artificial intelligence has yet to be developed; however, as seen in the Aquablation™, with appropriate human input and image planning, robots are capable of completing an operation autonomously. Shademan *et al*. recently published results from a porcine model with their Smart Tissue Autonomous Robot (STAR), which was able to complete the suturing of an intestinal anastomosis with better efficiency and consistency compared with laparoscopic and robotic surgeons ^41^. Opfermann *et al*. used the same system in a porcine model to resect squamous cell carcinoma pseudo-tumors marked with NIRF fiducials. The authors found that although resection by STAR took longer, it was able to achieve more consistently with the targeted resection than a human surgeon ^42^. Completely autonomous robotic surgeons with artificial intelligence are still in the realm of science fiction. However, it is clear that algorithms that combine artificial intelligence with image guidance and enhancement will allow surgeon-supervised autonomous surgery at some point in the not-too-distant future.

## Conclusions

The development of robotic platforms with urological applications in mind has resulted in the performance of minimally invasive surgeries once thought to be solely in the domain of open surgery. What is still uncertain is whether this rise in the utilization of robotics will be coupled with an inverse reduction in costs to health-care systems and improved outcomes and efficiencies for the masses. Although the technology has certain limitations, it is clear that there is ongoing interest in the development of solutions that will come to fruition as innovation and imagination lead the way.

SP robotic surgery will likely play an increased role in urology with the current push for less-invasive surgical techniques that reduce patient convalescence. There will also be an increased role for robotics in endoscopic procedures that will redefine how we think about these interventions, enabling mastery through technology. The next game-changing technology that will enable surgeons to perform operations more efficiently while improving outcomes is not far away and, with more competition, should result in driving down the significant expense associated with this technology.

## Abbreviations

3DHD, three-dimensional high-definition; AR, augmented reality; BPH, benign prostate hyperplasia; eGFR, estimated glomerular filtration rate; FDA, US Food and Drug Administration; FURS, flexible ureteroscopy; ICG, indocyanine green; IPSS, International Prostate Symptom Score; NIRF, near-infrared fluorescence; PET, positron emission tomography; PSM, positive surgical margin; PSMA, prostate-specific membrane antigen; RAPN, robot-assisted partial nephrectomy; RARP, robot-assisted radical prostatectomy; RLESS, robotic laparoendoscopic single site; SP, single-port; SS, single-site; STAR, Smart Tissue Autonomous Robot; TURP, transurethral resection of the prostate
